# Demonstration of Use of a Novel 3D Printed Simulator for Mitral Valve Transcatheter Edge-to-Edge Repair (TEER)

**DOI:** 10.3390/ma15124284

**Published:** 2022-06-17

**Authors:** Michele Bertolini, Michael Mullen, Georgios Belitsis, Angel Babu, Giorgio Colombo, Andrew Cook, Aigerim Mullen, Claudio Capelli

**Affiliations:** 1 Department of Mechanical Engineering, Politecnico di Milano, 20133 Milan, Italy; giorgio.colombo@polimi.it; 2 Barts Health NHS Trust, London E1 1BB, UK; mmullen@nhs.net; 3 Institute of Cardiovascular Science, University College London, London WC1E 6BT, UK; g.belitsis@ucl.ac.uk (G.B.); angel.babu.19@ucl.ac.uk (A.B.); a.cook@ucl.ac.uk (A.C.); a.mullen@ucl.ac.uk (A.M.); c.capelli@ucl.ac.uk (C.C.)

**Keywords:** mitral valve regurgitation, transcatheter edge-to-edge repair, MitraClip procedure, simulation-based training, segmentation, 3D printing, Polyjet

## Abstract

Mitral regurgitation is a common valvular disorder. Transcatheter edge-to-edge repair (TEER) is a minimally invasive technique which involves holding together the middle segments of the mitral valve leaflets, thereby reducing regurgitation. To date, MitraClip™ is the only Food and Drug Administration (FDA)-approved device for TEER. The MitraClip procedure is technically challenging, characterised by a steep learning curve. Training is generally performed on simplified models, which do not emphasise anatomical features, realistic materials, or procedural scenarios. The aim of this study is to propose a novel, 3D printed simulator, with a major focus on reproducing the anatomy and plasticity of all areas of the heart involved and specifically the ones of the mitral valve apparatus. A three-dimensional digital model of a heart was generated by segmenting computed tomography (CT). The model was subsequently modified for: (i) adding anatomical features not fully visible with CT; (ii) adapting the model to interact with the MitraClip procedural equipment; and (iii) ensuring modularity of the system. The model was manufactured with a Polyjet technology printer, with a differentiated material assignment among its portions. Polypropylene threads were stitched to replicate chordae tendineae. The proposed system was successfully tested with MitraClip equipment. The simulator was assessed to be feasible to practice in a realistic fashion, different procedural aspects including access, navigation, catheter steering, and leaflets grasping. In addition, the model was found to be compatible with clinical procedural imaging fluoroscopy equipment. Future studies will assess the effect of the proposed training system on improving TEER training.

## 1. Introduction

Mitral valve (MV) disorders are the most prevalent heart valve disease and the second most frequent condition requiring cardiac surgery [1,2,3,4]. Mitral regurgitation (MR) is the predominant heart valve disease in developed countries, found in 1.7% of the adult population [3,5]. It occurs due to the inability of the valvular leaflets to completely close during ventricular contraction. This leads to the retrograde flow of blood from the left ventricle (LV) into the left atrium (LA), decreasing the pumping efficiency of the heart towards the aorta [6].

Although surgical repair is still the gold standard treatment for this pathology, over the past years, an increasing number of transcatheter MV therapies have appeared [7]. A minimally invasive approach can reduce the procedural consequences on patients and therefore offer alternatives to those at high-risk in open-heart surgery [8,9]. Transcatheter edge-to-edge repair (TEER) is a minimally invasive procedure based on surgical edge-to-edge repair [10,11]. TEER is based on the implantation of a device which holds together the middle segments of the anterior and posterior mitral valve leaflets, thereby creating a double orifice mitral valve annulus which reduces the mitral regurgitant area. To date, MitraClip™ (Abbott Laboratories, Menlo Park, CA, USA) is the only US Food and Drug Administration (FDA)-approved device and has been implanted in more than 100,000 patients worldwide [12,13]. MitraClip is a cobalt-chromium catheter delivered device, which utilises two ‘grippers’ to grasp and coapt the MV leaflets. Recent clinical trials showed how such a procedure is comparable to, or more effective than, medical therapy [14,15].

From an operative point of view [16], a standard femoral venous access is required to perform a transseptal puncture (TSP) to deliver MitraClip steerable guide catheter (SGC) into the LA. The clip delivery system (CDS) is introduced through the SGC and positioned above the MV. During this process, it is vital for the operator to fully appreciate the spatial orientation of the interatrial septum (IAS), pulmonary veins (PV), left atrial appendage (LAA), MV leaflets, and subvalvular apparatus. Once in the LA, the clip must be positioned in the area of interest and oriented perpendicular to the line of MV coaptation. Then, the clip is advanced into the LV and grasping of the MV leaflets is performed with the open clip arms. When satisfactory insertion of the leaflets is confirmed, the clip is closed and deployed and the MitraClip delivery system is removed. The whole procedure is conducted under real-time 2D/3D Transoesophageal echocardiography (TOE) and X-ray fluoroscopy guidance [17].

The MitraClip procedure (MCP) is technically challenging [18]. The learning curve has been investigated by Eleid et al., who found that there was a sequential decrease in procedural time, but also in the number of adverse complications such as bleeding and in the length of post-operative hospital stay [19]. Training can therefore be crucial to shorten the learning curve and lead to better clinical outcomes in future.

Simulation-based training involves imitating a procedure’s scenario, so that clinicians are able to learn the procedural sequence in a more controlled situation, as well as receive feedback to help them improve [20]. Although simulators have now become widespread in clinical education, very few are the physical systems specifically designed for MCP. In this field, previous works showed examples of models of a whole heart [21] or specific portions [22,23] for practising the insertion of different transcatheter devices for the MV. However, to the best of our knowledge, anatomy-based systems presented so far do not allow the practising of the whole procedure including delivery, navigation, and device implantation.

This work introduces an anatomically and mechanically realistic training simulator, specifically developed for practising TEER procedure. Both design and manufacturing processes were approached in a multidisciplinary fashion, by means of an iterative process of feedback provided by experienced clinical end-users.

## 2. Materials and Methods

The study reports all the steps followed to create the novel simulator for TEER, including: (i) image segmentation; (ii) computed-assisted design modifications; (iii) selection of materials; and (iv) manufacturing by means of 3D printing technology. Finally, the use of the proposed simulator was demonstrated by practising a mock implantation of a MitraClip device. The study was approved by the local R&D office.

### 2.1. Image Segmentation

A stack of anonymised cardiac CT scans of adult subjects was used to generate the 3D digital model of the heart. The dataset consisted of 393 slices in the axial plane, with a resolution of 512 × 512 pixels. The size of each pixel was 0.35 mm, while the slice thickness was 0.75 mm. The commercially available Mimics software (Materialise, Leuven, Belgium, version 24) was used for segmentation. Atria and LV were segmented starting from the blood pool as independent masks, to guarantee better procedural flexibility in the design phases.

**Figure 1:**
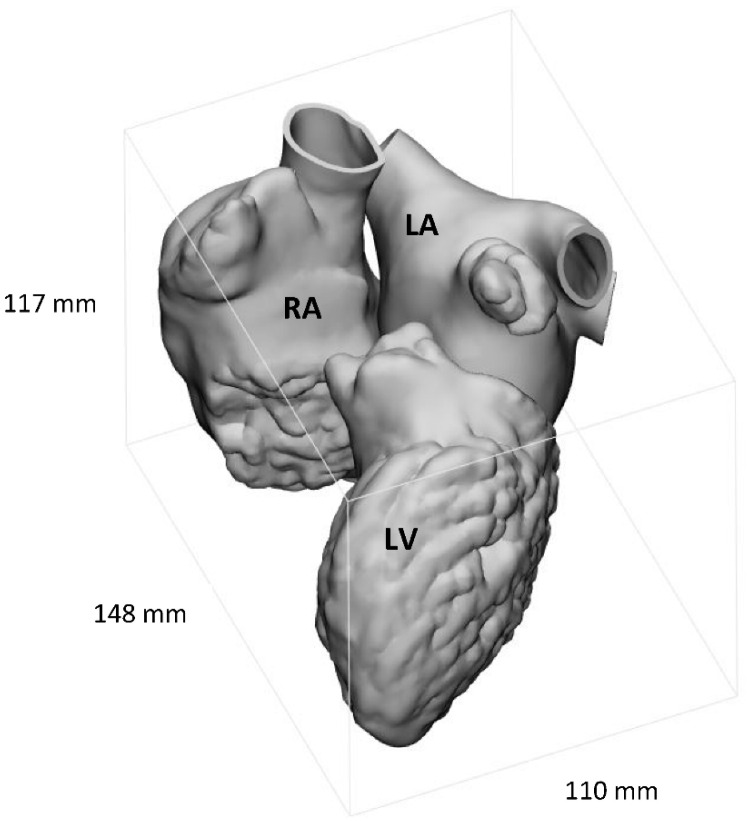
The reconstructed heart model portion, including right atrium (RA), left atrium (LA), and left ventricle (LV). The bounding box with real dimensions (expressed in millimetres) is highlighted.

Each mask was converted into STL files and exported into 3-Matic (Materialise, Leuven, Belgium, version 15) for post-processing operations, namely wrapping, smoothing, and mesh fixing. The result is shown in Figure 1.

### 2.2. Computed-Assisted Design Modifications

The heart model was subsequently modified for (i) adding anatomical features not entirely visible with CT; (ii) adapting the model to interact with the MitraClip system; and (iii) ensuring modularity of the system.

#### 2.2.1. Additional Anatomical Features

**Figure 2:**
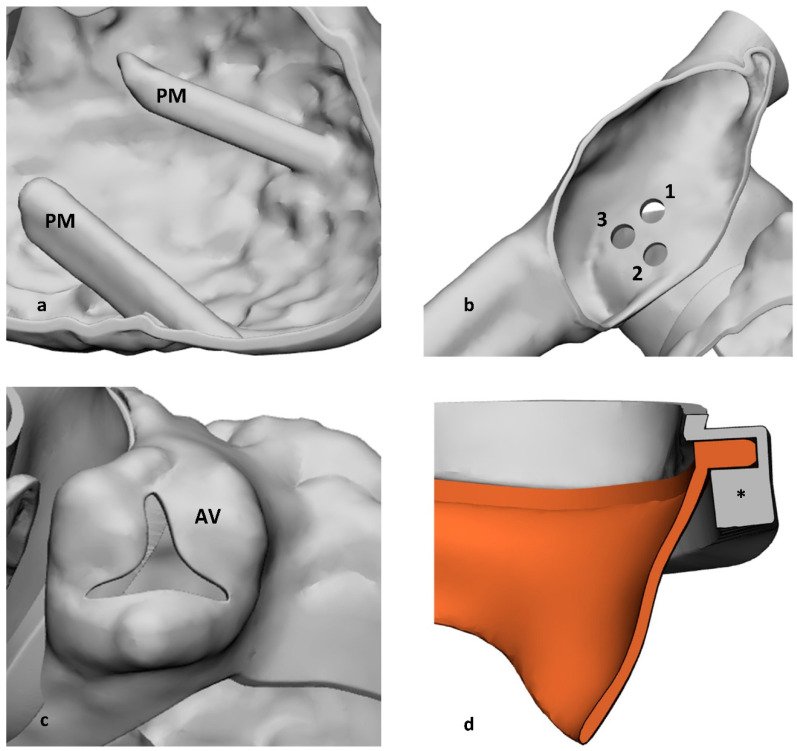
Details of the computed-assisted design modifications of the model. (**a**): Extruded papillary muscles (PM). (**b**): Transseptal holes to practice different approaches of the puncture (1—posterior-superior; 2—anterior-inferior; 3—posterior-inferior). (**c**): Aortic valve (AV). (**d**): Cross sectional view of the modular insertion of the MV annulus (*) and MV leaflets (orange).

Papillary muscles (PMs) were not entirely visible with medical imaging. Only their tips could be clearly identified in the segmentation phase. The rest of the muscles were created by extruding the cross-section of the tips onto the LV wall (Figure 2a). Three round-shaped 22 Fr transseptal holes were created in the IAS (Figure 2b) in order to simulate the three most common locations of transeptal puncture through the oval fossa: position 1—in a posterior-superior aspect, i.e., the optimal TSP; position 2—anterior-inferior, i.e., to simulate so-called “aorta-hugging” scenario; position 3—posterior-inferior puncture, i.e., another sub-optimal approach. The commissures of the aortic valve (AV) leaflets were marked to provide a landmark for orientation (Figure 2c).

#### 2.2.2. Adaptation of the Model to MCP

Openings were inserted in the models to allow a clear view of the implantation site during the simulated procedure: the right ventricle (RV) with interventricular septum (IVS), vestibular part of RA, and posterior wall and the roof of the LA were removed. The inferior vena cava (IVC) was extended by 40 mm, in order to facilitate the insertion of the catheter.

**Figure 3:**
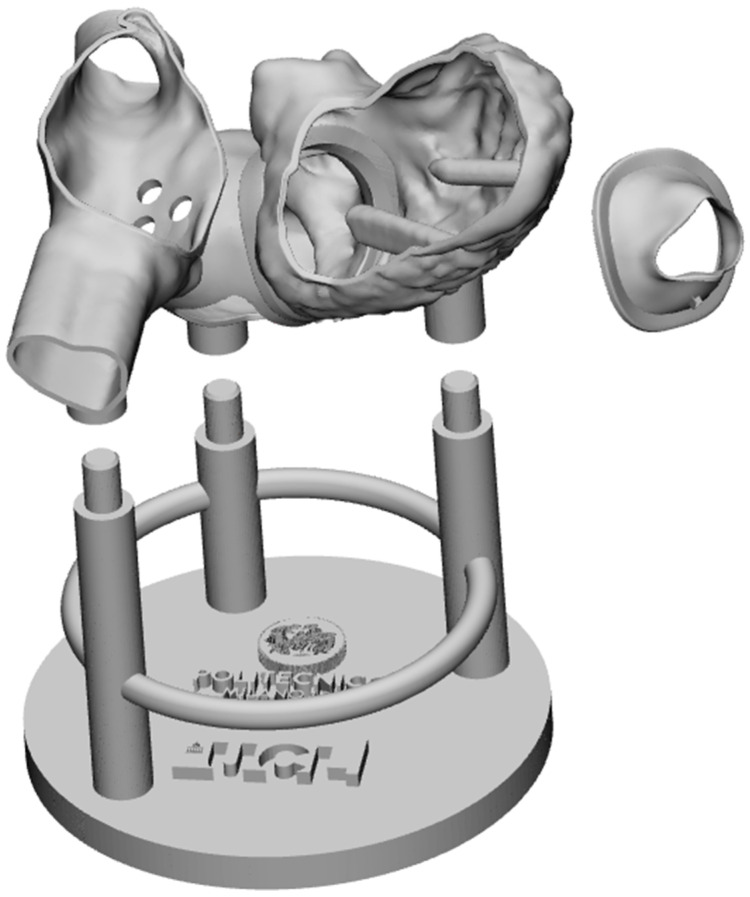
The digital model of the simulator, shown in an exploded view.

A basement for the anatomical part was designed to guarantee the stability of the model in a position that replicates the heart of the patient on the operating table. A round plate with three columns, interconnected by a toroid, was designed (Figure 3). To guarantee the coupling with the anatomical part, three chamfered cylinders were added to the top of each column.

#### 2.2.3. System Modularity

The MV leaflets were designed to be replaceable. This was achieved by a geometric matching between the MV annulus and the atrioventricular wall. The MV leaflets were included in a mid-systole configuration. By means of the sweep feature, an insertion slot was created. A 0.1 mm gap between annulus and slot was left for the matching (Figure 2d). Two pinholes were placed along the ring for an extra fixation between valve and ring by means of threads. The position of these holes on the ring was chosen to offer proper resistance to excessive pulling of the composite valve during the application of the MitraClip. The ring was then merged, by means of a Boolean union, to the rest of the heart.

The full design with its modular components is shown in Figure 3.

### 2.3. 3D Printing and Assembling

The model was fabricated by means of 3D printing technology. The cardiac model was manufactured by means of a Polyjet Stratasys J835 printer (Stratasys, Eden Prairie, MN, USA), while for the basement, an SLS EOS Formiga P100 (EOS, Krailling, Germany) was used. The cardiac model was printed with two different blends, made of commercial Stratasys VeroClear (rigid) and Agilus30 Clear (soft) [24]. The hardness of choice was selected through a qualitative but systematic evaluation of the mechanical behaviour on cylindrical specimens (outer diameter 20 mm, thickness 1.6 mm), printed varying the resulting shore A hardness (40 A, 50 A, 60 A, 70 A, 85 A, 95 A, and RGDA8630-DM blend). Three clinicians of our group were asked to select the specimen which could provide the most similar feedback as based on their experience.

The selected stiffer blend, coded RGDA8630-DM, was used for the ring housing the valve, to guarantee stable positioning, and for the cylinders connecting the model to the base. Atria and LV walls were printed with a combination of VeroClear and Agilus resulting in a shore hardness of 85 A. MV leaflets were printed with pure Agilus30. The basement was built with a PA2200 polymeric powder. Polyjet models had to be soaked in soda solution to facilitate support structure removal. 

MV leaflets were mounted within the slot of the ring and supported by fine stitches. A replica of chordae tendineae was also inserted in the model, by means of polypropylene (PP) sutures between the distal tips of MV and PMs heads. Their insertion on this model was performed by the surgeon of our team (GB), after sizing the leaflet and defining a coaptation height of 7 mm [25]. The chordae tendineae were inserted while the valve was kept apposed centrally and in a fashion that the superior and inferior PMs supported both leaflets at the corresponding commissures, replicating the functional anatomy of the valve.

### 2.4. Testing

The prototype of the anatomical model was assessed to verify the feasibility of the MCP. Two mock procedures were carried out both outside and inside the catheterisation laboratory (Cath lab). The testing was performed by an expert MitraClip implanter (MM, total experience of over 100 cases of TEER), following all the steps detailed here.

I. Preparation. The stabiliser of the MitraClip system was positioned about 80 cm away from the model, ensuring sufficient distance for TSP. The base of the model was stabilised on the table.
II. Guide positioning in the LA and introducing CDS. The steerable guide catheter was advanced through the IVC, then transseptal crossing passage and insertion of CDS through SGC. A series of steering manoeuvres with the SGC and CDS were made to optimise the trajectory and orientation of the clip arms perpendicular to the line of coaptation, under direct visual access through the openings of the model.
III. Advancing into LV through the MV while avoiding interaction with subvalvular apparatus and leaflet grasping. Once in place, MV leaflets were grasped by lowering the grippers and closing clip arms. Qualitative and visual assessments of MR were performed, possibly accompanied by leaflets regrasping, to reach optimal positioning and sufficient grasping.
IV. Deployment simulation and system removal. CDS was removed by releasing deflection on the catheter and slowly removing it from the model.

The second test of the mock implantation was performed under standard cardiac monoplane or biplane Cath lab. This test was performed with the fluoroscopy equipment available at St. Bartholomew’s Heart Centre (Azurion 7, Philips NV, Amsterdam, The Netherlands) with rotation and tilting an antero-posterior (AP) arm of the fluoroscopy system in order to achieve a desirable angulation of the cardiac structures on the screen. The simulator was placed in a standard projection on the operating table under the AP fluoroscopy arm and the MitraClip kit was installed 80 cm away, at the caudal end of the table.

## 3. Results

**Figure 4:**
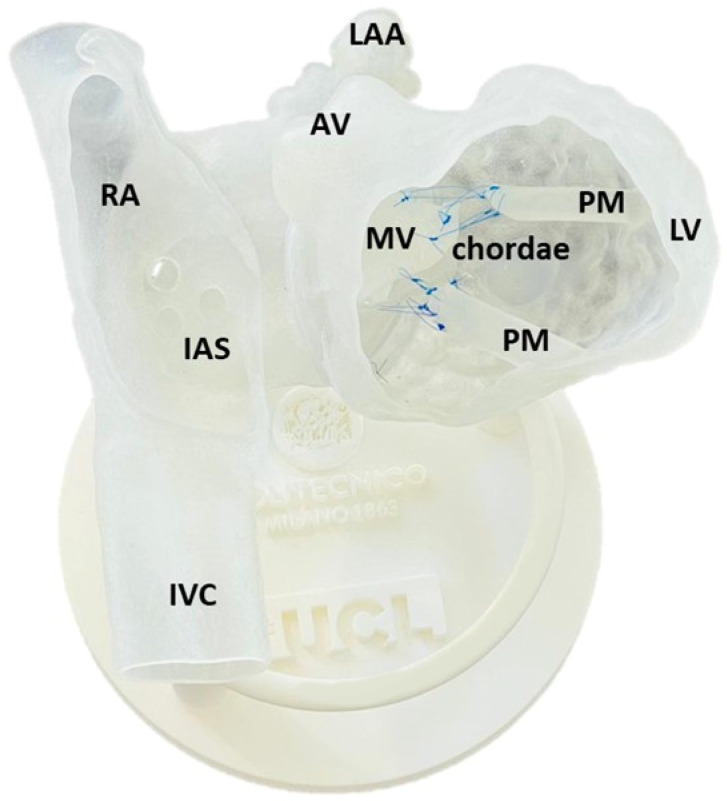
The 3D printed simulator assembled to practice a mock intervention of TEER. Labels indicate the main anatomical features: aortic valve (AV), chordae, interatrial septum (IAS), inferior vena cava (IVC), left atrial appendage (LAA), left ventricle (LV), mitral valve (MV), papillary muscles (PM), and right atrium (RA).

The ready-to-use simulator with stitched chordae tendineae is shown in Figure 4. Total printing time was about 20 h. A total of 450 g of resins were used for the model, together with 850 g of support material.

The model was an accurate representation of the anatomical details, as per [24], and characterised by a satisfactory haptic sensation for the material and optimal finishing. The matching between model and base and between model and interchangeable MV was seamless. At the transition from a material region to another, no sharp distinction was notable, suggesting excellent integration between parts.

**Figure 5:**
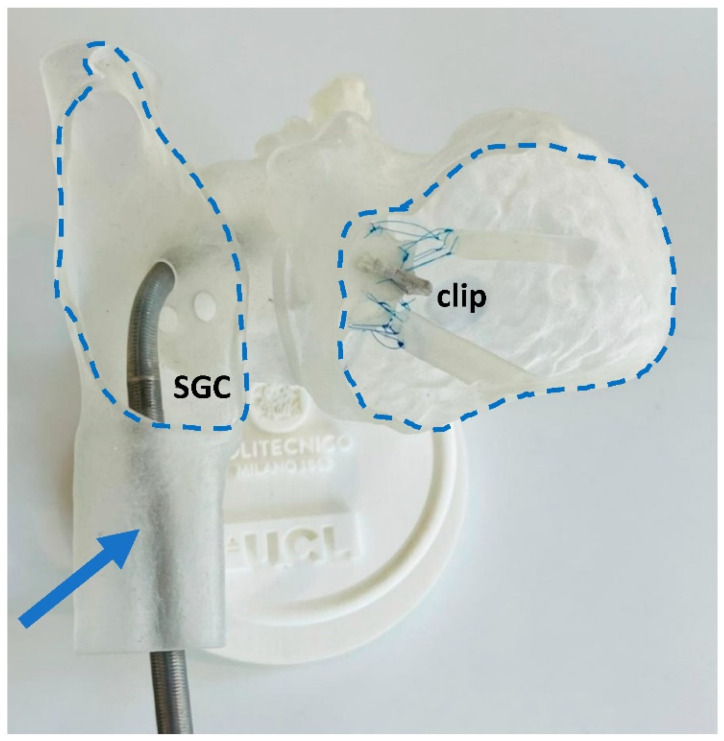
The steerable guide catheter (SGC) is visible in the printed model. The blue arrow identifies it within the IVC, while the dashed lines show the designed windows for visualisation from outside.

The prototype was translucent, allowing the operator to see through the walls and track the position of the SGC when deployed (Figure 5). This, together with the presence of ad hoc windows opened in the model, allows the operators a full view of all the anatomical structures involved in the TEER.

**Figure 6:**
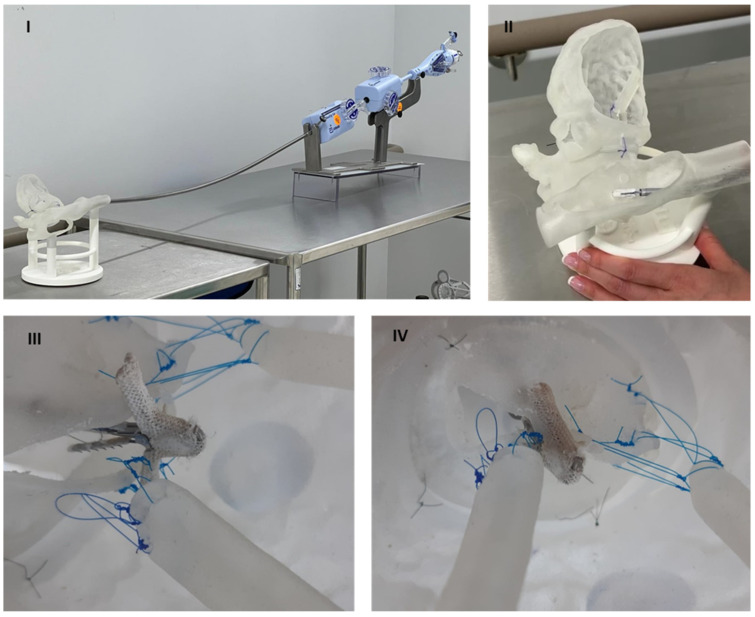
Report of the steps of the MitraClip simulator testing procedure. (**I**): Preparation. (**II**): Guide insertion. (**III**): Positioning of the clip. (**IV**) Implantation.

The mock procedure of TEER was successfully achieved. Figure 6 shows the four procedural steps: preparation, guide insertion, positioning of the clip, and implantation.

**Figure 7:**
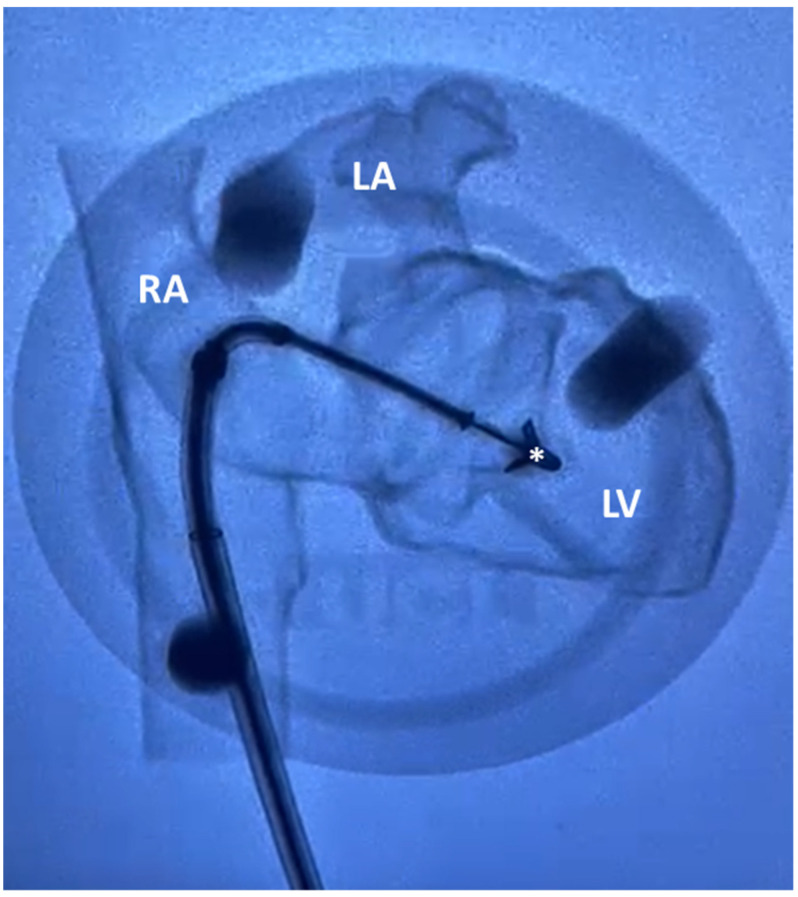
The 3D printed simulator, with deployed CDS, visualised under fluoroscopic imaging. Left atrium (LA), left ventricle (LV), right atrium (RA) and the clip (*) are labelled.

The TEER was successfully repeated under fluoroscopy guidance (Figure 7). During X-ray screening the view of a contour of the 3D printed model corresponded to that in a patient. As for the previous simulation, the mock implantation started from SGC insertion into IVC, crossing the TSP, advancing CDS to the LA cavity, then navigating and positioning the clip above the MV. Clip orientation was performed under visual control of the proctor (mimicking Echo guidance) and confirmation with fluoroscopy images by implanter. The clip was then advanced to the LV cavity and MV leaflet grasping was tested with success.

## 4. Discussion and Conclusions

In this work, a novel training simulator for practising TEER procedures was designed, manufactured, and successfully tested with MitraClip equipment. Starting from CT images, the geometry of a 3D model was generated and augmented further so the end result realistically replicated both the pathway for the TEER delivery system and the MV functional anatomy. The presented simulator was compatible to be used for training TEER procedures.

This model intends to advance the field of TEER training simulators as it includes anatomical details which can challenge the operator in performing the procedure (i.e., three different approaches for transeptal punctures, interchangeable leaflets, replica of chordae tendineae and papillary muscles). Such details have been neglected in previous simulators, as reported in the introduction. In addition, we have reported a design process which takes into account the overall training experience. In this context, on one hand, the materials were chosen to guarantee a realistic haptic response from the operators; on the other hand, the model includes openings specifically designed to guarantee full visual assessment of the different phases of the implantation procedure.

This study reports the first usages by experienced clinicians. The results of the feasibility assessment were fully satisfactory. We have shown how the proposed model is fully compatible with the MitraClip equipment. The users appreciated the anatomical accuracy together with the possibility of being fully aware of the spatial orientation in all the different phases of the implantation procedures. Other recognised strengths of this simulator included the modularity of the system which allows the replacement of single components in case of damages or tears, the low cost, and the mechanical response. This also guarantees the possibility of changing valve geometries in order to test potentially different patients’ configurations or different phases of the cardiac cycle.

The preliminary tests reported here show the possibility of using the simulator in the Cath lab. The radiopacity of the model was confirmed under fluoroscopy setting. This is particularly relevant to replicate the setting of the real experience. Fluoroscopy is a compulsory imaging component during the TEER procedure. The test showed how the proposed 3D printed anatomical model allows a trainee to identify the location and borders of the actual cardiac structures and obtain a skill of manipulating the MitraClip system, without direct access to the training model but based on the fluoroscopy images and haptic feedback. Hence, the procedural training can become closer to real-life experience.

The pilot nature of this study implies some limitations. One of the main ones is related to the qualitative evaluations of the mechanical properties of the printed model. Our choice was mainly based on the feedback received by operators experienced in TEER. TEER is a minimally invasive procedure and the navigation through the cardiac structures is based both on imaging and haptic feedback as a result of the interaction between delivery system and anatomical structures (i.e., heart and surrounding organs). Replicating this feedback for training purposes was at the core of our project. Hence, in this study, we decided to take the route of a qualitative, experienced assessment. A direct comparison between the model’s materials and biological tissues would be also possible [26,27]. However, this could provide information on the anatomical component in isolation rather than consider the response of the system.

The basement was designed to keep the anatomical model stable, oriented in a position compliant with the normal conditions and at a fixed distance from the operator. All these factors enabled us to have controlled and repeatable conditions of the training. However, quantitative values regarding the stress conditions experienced during the transcatheter procedure are not available in the literature. For this reason, even in this case we had to rely on the experience of clinical users to have a validation of the simulator behaviour. A more rigorous evaluation of this aspect could be part of future studies.

The design of the simulator can be further improved to include movable leaflets [28], to further increase the realism of the training, especially in relation to MV leaflets grasping. The presented concept of 3D printed could be also extended to different and challenging transcatheter interventions such as, for example, mitral valve replacement or interventions on the tricuspid valve, for which there is a rise in percutaneous therapies [29,30,31].

Lastly, the benefit of using such a simulator to practice TEER will need to be extensively investigated and compared to existing solutions. Collected feedback by clinicians, although highly satisfactory, are not conclusive. A quantitative, statistical analysis will be the object of a future study, in which the performance of the simulator will be assessed by testing multiple operators and compared to the state-of-the-art solution.

In conclusion, this study reports the design and preliminary testing of a new 3D printed simulator for TEER of regurgitant mitral valves. The prototype was tested to be fully compatible with MitraClip equipment, demonstrating the feasibility of the training. Future studies will systematically explore the benefits of the proposed new design as compared to existing solutions.

## Data Availability

Not applicable.
